# Manejo integrado de tuberculose e diabetes: uma revisão integrativa

**DOI:** 10.26633/RPSP.2019.21

**Published:** 2019-02-06

**Authors:** Cíntia Vieira Nascimento, Sônia Maria Soares

**Affiliations:** 1 Hospital Júlia Kubitschek Hospital Júlia Kubitschek Fundação Hospitalar do Estado de Minas Gerais (FHEMIG) Belo HorizonteMG Brasil Fundação Hospitalar do Estado de Minas Gerais (FHEMIG), Hospital Júlia Kubitschek, Belo Horizonte (MG), Brasil.; 2 Escola de Enfermagem Escola de Enfermagem Universidade Federal de Minas Gerais Belo HorizonteMG Brasil Universidade Federal de Minas Gerais, Escola de Enfermagem, Belo Horizonte (MG), Brasil.

**Keywords:** Tuberculose, diabetes *mellitus*, diagnóstico, monitoramento epidemiológico, América Latina, Tuberculosis, diabetes *mellitus*, diagnosis, epidemiological monitoring, Latin America, Tuberculosis, diabetes *mellitus*, diagnóstico, monitoreo epidemiológico, América Latina

## Abstract

**Objetivo.:**

Identificar as evidências sobre manejo integrado de tuberculose e diabetes disponíveis na literatura para o contexto latino-americano.

**Métodos.:**

Foi realizada uma revisão integrativa da literatura com busca nas bases de dados LILACS, Web of Science e PubMed. A estratégia utilizou como termos de busca “tuberculose”, “diabetes mellitus” e “manejo integrado”. Foram incluídos artigos científicos sobre estudos realizados na América Latina, publicados de 2011 a 2017, com acesso livre ao texto integral e publicação em inglês, espanhol ou português. Foram coletados dados relativo aos autores, delineamento, amostra, principais resultados, país e ano da publicação. Finalmente, os estudos foram classificados em níveis de evidência.

**Resultados.:**

Foram incluídos 20 estudos, dos quais 60% apresentaram baixa evidência científica (nível IV). Conforme esses estudos, os pacientes com diabetes possuem maior risco de desenvolver tuberculose, especialmente aqueles com controle glicêmico ineficaz. Além disso, pacientes com tuberculose-diabetes apresentam atraso na conversão do escarro e maior probabilidade de falha terapêutica e morte. Há maior prevalência da associação tuberculose-diabetes no sexo masculino. Em relação aos registros de tuberculose e diabetes em prontuários ou fichas eletrônicas de informação nos serviços de saúde, há falhas ou ausência de anotações. Foi identificado alto custo financeiro do manejo clínico em indivíduos com a comorbidade. A equipe multidisciplinar possui papel imprescindível na prevenção e promoção em saúde, nos três níveis de atenção.

**Conclusão.:**

O rastreamento bidirecional de tuberculose-diabetes poderá implicar em melhor controle desses agravos, principalmente em países em desenvolvimento e em áreas endêmicas para tuberculose.

## Abstract (en)

**Objective.:**

To identify the evidence regarding the co-management of tuberculosis and diabetes available in the literature for the Latin-American context.

**Method.:**

An integrative literature review was performed in the LILACS, Web of Science, and PubMed databases. The search strategy employed the terms “tuberculosis”, “diabetes mellitus,” and “integrated management.” Open access articles reporting studies performed in Latin America, published from 2011 to 2017 in English, Spanish or Portuguese were included. The following information was collected: authors, design, sample, main results, country of study, and year of publication. The studies were also classified according to level of evidence.

**Results.:**

Twenty studies were included, 60% of which had low level of evidence (level IV). According to these studies, patients with diabetes are at increased risk for tuberculosis, especially those with poor glycemic control. In addition, tuberculosis-diabetes patients have delayed sputum conversion and higher probability of treatment failure and death. The prevalence of tuberculosis-diabetes comorbidity is higher in males. Information about tuberculosis and diabetes is poorly recorded or absent in medical charts or electronic files. The management of tuberculosis-diabetes comorbidity is associated with high cost. The multidisciplinary health care team plays a crucial role in the prevention and promotion of health at all levels of care.

**Conclusion.:**

Bidirectional screening of tuberculosis-diabetes may facilitate the control of these disorders, especially in developing countries and endemic tuberculosis areas.

## RESUMEN

**Objetivo.:**

Identificar la evidencia científica disponible en la bibliografía sobre el manejo integrado de la tuberculosis y la diabetes en el contexto de América Latina.

**Métodos.:**

Se realizó una revisión integrativa de la bibliografía con búsqueda en las bases de datos LILACS, Web of Science y PubMed. Se efectuó una búsqueda empleando los términos “tuberculosis”, “diabetes mellitus” y “manejo integrado”. Se incluyeron artículos científicos de estudios realizados en América Latina, publicados desde 2011 hasta 2017, disponibles en acceso libre y texto completo en inglés, español o portugués. La información recolectada incluyó datos relativos a los autores, diseño, muestra, resultados principales, país y año de la publicación. Finalmente, se clasificaron los estudios según los niveles de evidencia.

**Resultados.:**

Se incluyeron 20 estudios, de los cuales el 60% presentó evidencia científica baja (nivel IV). Según estos estudios, los pacientes con diabetes tienen mayor riesgo de desarrollar tuberculosis, especialmente aquellos con un control ineficaz de la glucemia. Además, los pacientes con tuberculosis y diabetes presentan un retraso en la conversión del esputo y una mayor probabilidad de fracaso terapéutico y de morir. En el sexo masculino existe mayor prevalencia de la asociación de tuberculosis y diabetes. En cuanto a los registros de tuberculosis y diabetes en las historias clínicas o fichas electrónicas de información en los servicios de salud, hay deficiencia o falta de anotaciones. Se identificó un alto costo financiero relacionado con el manejo clínico en los individuos con ambas enfermedades. El equipo multidisciplinario tiene un papel imprescindible en la prevención y la promoción de la salud en los tres niveles de atención.

**Conclusión.:**

El rastreo bidireccional de la tuberculosis y la diabetes puede implicar un mejor control de estas enfermedades, principalmente en los países en desarrollo y en las áreas endémicas para la tuberculosis.

A tuberculose ainda é considerada uma doença negligenciada, sendo responsável pela contaminação de milhões de pessoas a cada ano. Representa um problema de saúde global, ocupando a segunda colocação nas causas de morte por doenças infecciosas no mundo. Em 2015, de 10,4 milhões de pessoas com tuberculose, 1,4 milhão morreram (1). Nesse cenário epidemiológico, também causa preocupação o aumento das condições crônicas, como o diabetes. Em 2013, o número estimado de pessoas com diabetes chegou a 382 milhões, projetado para alcançar 592 milhões em 2035 (2).

Estudos demonstram que o diabetes – por ser uma doença crônica, que enfraquece o sistema imunológico – é uma ­ameaça ao controle mundial da tuberculose (3-6), aumentando o risco geral de infecção, fazendo com que seus portadores possuam uma probabilidade três vezes maior de contrair tuberculose ativa (1, 6). Em pessoas que iniciaram tratamento para tuberculose, o diabetes está associado com demora na conversão do bacilo álcool-ácido resistente (BAAR) e aumento do risco de reinfecção ou óbito, mesmo após tratamento ­adequado (6).

Tendo em vista a preocupante associação entre tuberculose e diabetes, a Organização Mundial da Saúde (OMS) e a União Internacional contra Tuberculose e Doenças Pulmonares (*International Union Against Tuberculosis and Lung Disease*, UNION) elaboraram diretrizes internacionais para a gestão conjunta dessas comorbidades. As duas organizações publicaram, em 2011, um quadro de recomendações (7) no qual definem as principais alternativas para o rastreamento bidirecional e a gestão coordenada das duas doenças nos serviços de saúde, com detecção e controle do diabetes em pacientes com tuberculose e detecção e controle da tuberculose em pacientes com diabetes. A ­associação entre esses agravos acarreta para o sistema de saúde maior tempo de internação, maior custo de tratamento, principalmente devido ao aumento da prevalência de tuberculose multidrogarresistente (TBMDR), e maior taxa de mortalidade. Além disso, a população acometida encontra-se em idade produtiva; uma recuperação mais rápida e um menor tempo de internação permitem um retorno mais precoce ao trabalho, trazendo menos efeitos econômicos negativos para pacientes e famílias de baixa renda (3, 6, 9).

Estima-se que, até 2030, 80% dos pacientes com diabetes vivam na América Latina, sendo importante compreender as características clínicas e epidemiológicas do binômio tuberculose-diabetes nessa região para tratamento e controle adequados (10). Portanto, o objetivo do presente estudo foi identificar as evidências sobre manejo integrado de tuberculose e diabetes disponíveis na literatura para o contexto latino-americano.

## MATERIAIS E MÉTODOS

Foi realizada uma revisão integrativa da literatura com propósito de sintetizar os principais resultados de estudos acerca da associação tuberculose-diabetes no contexto latino-americano. A revisão envolveu as seguintes etapas: estabelecimento do problema; seleção da amostra; caracterização dos estudos; e análise, discussão e apresentação dos resultados.

Para a busca dos artigos, formulou-se a seguinte questão: quais recomendações técnicas e cuidados contemplam o manejo integrado dos pacientes com tuberculose e diabetes? A estratégia de busca foi construída a partir dos descritores em ciências da saúde (DeCS) “tuberculose” e “diabetes *mellitus*”, com as seguintes combinações “diabetes and tuberculosis” ou “diabetes e tuberculose”*.* Também foi utilizado o termo “manejo integrado”, que não é um DeCS, mas se refere à temática central deste estudo e é bastante utilizado na literatura. A seleção foi composta por publicações indexadas nas bases de dados LILACS (http://lilacs.bvsalud.org/), PubMed (https://www.ncbi.nlm.nih.gov/pubmed/) e Web of Science (https://www.periodicos.capes.gov.br/).

Os critérios de inclusão foram: ser artigo científico sobre estudo realizado na América Latina, publicado de 2011 a 2017; estar disponível na íntegra com acesso livre; e ter sido publicado em inglês, espanhol ou português. O ano inicial de busca foi definido em função de ter sido esse o ano de publicação das recomendações para gestão combinada das duas doenças (7). Foram excluídos artigos que não contemplavam as recomendações da OMS, que foram realizados em outras regiões geográficas e documentos não oficiais.

**FIGURA 1:**
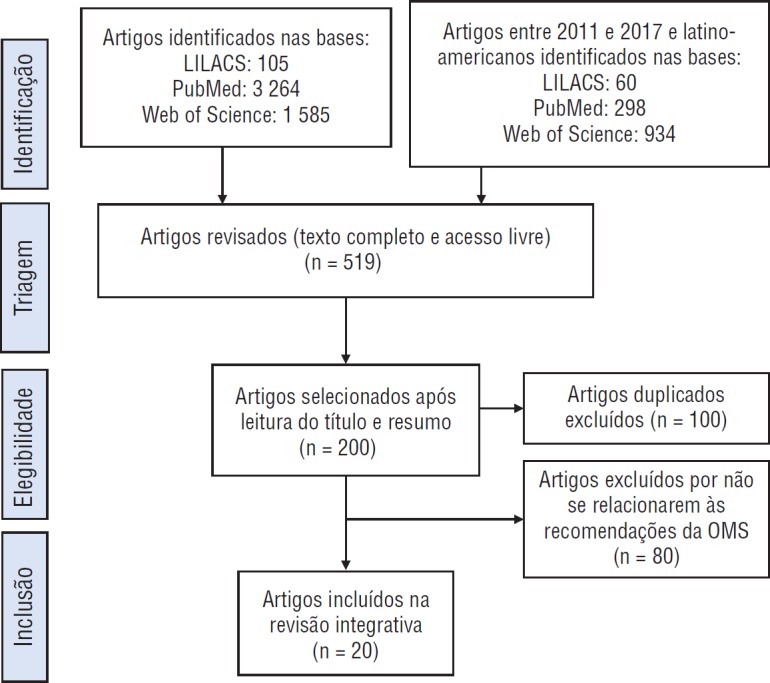
Processo de seleção de artigos na revisão sobre manejo integrado de tuberculose e diabetes

A busca de dados ocorreu de janeiro a fevereiro de 2018. A seleção dos artigos envolveu inicialmente a avaliação de títulos e resumos e a seleção de artigos para leitura integral. Desses, foram eliminados os que não abordavam o manejo integrado conforme as recomendações da OMS e os artigos duplicados. A figura 1 mostra o processo de seleção de artigos.

Para síntese da coleta de dados foram contemplados os seguintes itens: autores, delineamento do estudo, amostra, principais resultados, país e ano da publicação. Finalmente, os estudos foram classificados em níveis de evidências, conforme proposto por Galvão (11).

## RESULTADOS

A partir da seleção, obtiveram-se 20 artigos (figura 1). Quanto ao nível de evidência, foram incluídos três estudos de nível I (revisão sistemática ou metanálise de múltiplos estudos controlados ou diretrizes clínicas com revisão sistemática), um estudo de nível II (ensaio clínico randomizado), um estudo de nível III (ensaio clínico bem delineado e não randomizado), 12 estudos de nível IV (coortes e estudos de caso-controle bem delineados) e três estudos de nível VI (descritivos ou qualitativos). O nível V (revisão sistemática de estudos descritivos e qualitativos) não foi encontrado.

Em relação à distribuição geográfica, o Brasil liderou as publicações, com 10 estudos; outros cinco foram realizados no Peru, três no México e dois no Chile. A tabela 1 resume os principais achados.

### Prevalência de comorbidade

A prevalência de comorbidade tuberculose-diabetes foi informada em 12 estudos, sendo as duas maiores taxas registradas no México – 30% e 19% dos pacientes, respectivamente (12, 13). As menores prevalências foram no Brasil, de 3,7% e 3,8%, respectivamente (14, 15). Essa diferença marcante entre as prevalências pode ser compreendida pela forma como os dados foram coletados: no México, o rastreio do diabetes foi realizado através de dados primários, coletados pelo monitoramento da glicemia capilar e da hemoglobina glicada (HbA1c) durante a internação do paciente; já nos estudos brasileiros, as informações foram obtidas de dados secundários, registrados no Sistema de Notificação e Controle de Agravos (SINAN).

**TABELA 1:** Síntese de artigos publicados de 2011 a 2017 sobre o manejo integrado de tuberculose e diabetes no contexto latino-americano

Autor/referência	Delineamento/nível de evidência	Amostra/objetivo	Principais resultados	País/ano
Miranda (36)	– Revisão sistemática – Nível I	O objetivo foi revisar o tratamento da tuberculose em situações especiais no Brasil: pacientes com hepatopatias, nefropatias, diabetes, gestantes, nutrizes e soropositivos para o HIV.	O tratamento da tuberculose em pacientes com diabetes é o esquema básico. Porém, para aqueles que usam hipoglicemiante oral, deve-se aumentar a dose e realizar um controle rigoroso da glicemia, que deve ser mantida abaixo de 160 mg/dL. O Ministério da Saúde orienta a troca do hipoglicemiante oral por insulina para um melhor controle da glicemia, devido à interação com a rifampicina. O tempo de tratamento de tuberculose para os pacientes com diabetes em uso de insulina pode ser prolongado.	Brasil 2012
Arnold et al. (28)	– Revisão sistemática – Nível I	84 estudos sobre a associação entre tuberculose e diabetes no mundo e sobre aspectos clínicos e epidemiológicos.	A resistência a drogas antituberculose é comum em pessoas com diabetes. Existe uma relação entre hiperglicemia e aumento da susceptibilidade à infecção por tuberculose. A mortalidade está entre 4% e 8%.	Peru 2012
Gil e Moore (27)	– Revisão sistemática – Nível I	45 estudos para informar as linhas de pesquisa que estão sendo desenvolvidas para entender o problema da associação entre tuberculose e diabetes.	Devido ao aumento do risco de tuberculose ativa em doentes com diabetes, a falta de gestão adequada do controle da tuberculose na população com diabetes traz impacto negativo para todo o sistema de saúde.	Peru 2014
Castellanos-Joya et al. (9)	– Ensaio clínico randomizado – Nível II	– 783 sintomáticos respiratórios rastreados para tuberculose de 7 763 pacientes com diagnóstico prévio de diabetes. – 361 (40,8%) de 885 pacientes com tuberculose foram rastreados para diabetes.	Diagnóstico de tuberculose em 38 (4,9%); 11 (28,9%) não tinham conhecimento da doença. Diabetes foi diagnosticado em 70 (19,4%); 16 (22%) não tinham conhecimento da doença. Diagnóstico da comorbidade em 108 (1,24%) pacientes.	México 2014
Herrera et al. (18)	– Estudo analítico transversal – Nível III	– Todos os casos de tuberculose acima de 15 anos da região metropolitana do Chile em 2012 constantes no Registro Nacional de Tuberculose foram pesquisados ​​no Sistema de Informação para determinar a presença de diabetes.	Dos 821 casos de tuberculose na região metropolitana, a prevalência de diabetes foi de 15,6%. A taxa de incidência estimada de tuberculose entre a população diabética foi de 24,3 por 100 000, 1,7 vez a da população geral da região.	Chile 2012
Ramonda et al. (20)	– Estudo de caso-controle – Nível IV	– Estabelecer a relação entre diabetes e tuberculose para justificar a localização de casos em diabéticos com sintomas respiratórios, utilizando 473 casos e 507 controles.	O diabetes está associado a um risco aumentado de contrair tuberculose, com implicações clínicas e epidemiológicas. Cerca de 80% das pessoas com diabetes sabem que estão doentes.	Chile 2012
Reis Santos et al. (15)	– Estudo de coorte retrospectivo – Nível IV	– Avaliar as diferenças sociodemográficas e clínicas em 29 275 indivíduos com tuberculose com diabetes (n = 1 797) e sem diabetes usando o sistema nacional de vigilância.	A prevalência de diabetes foi de 5,4%. Indivíduos com comorbidade tendiam a ser mais velhos; ter mais comorbidades; ter teste de esfregaço de escarro positivo inicial e maior mortalidade.	Brasil 2013
Jiménez et al. (12)	– Estudo de coorte prospectivo – Nível IV	– 1 262 pacientes com tuberculose rastreados para diabetes de março de 1995 a abril de 2010	29,63% foram diagnosticados com diabetes antes do diagnóstico de tuberculose. Esses apresentaram maior probabilidade de serem do sexo feminino, idosos e de nível socioeconômico mais elevado.	México 2013
Magee et al. (19)	– Estudo de coorte prospectivo – Nível IV	– De 2005 a 2008, 1 671 adultos suspeitos de tuberculose receberam testes rápidos de sensibilidade a drogas e foram prospectivamente matriculados e seguidos durante o tratamento.	186 (11,1%) adultos com tuberculose tiveram diabetes. A prevalência de tuberculose resistente a múltiplos medicamentos foi de 23% entre não diabéticos e 26% entre os sem diabetes. Dos 149 pacientes com comorbidade, 104 (69,8%) apresentavam tuberculose sensível e 45 (30,2%) tuberculose resistente a drogas.	Peru 2013
León (25)	– Estudo retrospectivo observacional – Nível IV	– 1 083 pacientes com tuberculose de 2010 a 2012 foram rastreados para diabetes, controle glicêmico e sensibilidade aos medicamentos.	7,3% tinham diabetes associado; em 79,7% (n = 63), o controle glicêmico era ruim; 45,6% (n = 36) apresentavam resistência a um ou mais fármacos antituberculose.	Peru 2014
Reis-Santos et al. (14)	– Estudo de prevalência – Nível IV	– Avaliar fatores sociodemográficos e clínicos que podem influenciar os diferentes resultados dos 990 017 pacientes com tuberculose identificados na base de dados de 2001 a 2011 e analisar a prevalência de diabetes.	36 920 dos indivíduos com tuberculose tinham diabetes. Apesar de um declínio geral na incidência de tuberculose, os diabéticos aumentaram substancial e progressivamente entre os tuberculosos de 2001 a 2011. A taxa de cura encontrada foi de 80%.	Brasil 2014
Guadalupe et al. (13)	– Coorte retrospectiva transversal – Nível IV	– Análise das tendências de incidência de comorbidades de tuberculose e diabetes e resultados de tratamento de acordo com o diagnóstico em 181 384 pacientes (94,51%) com diabetes no período de 2000 a 2012.	A frequência de diabetes entre os pacientes com tuberculose foi de 19,29%. Pacientes com tuberculose pulmonar com diagnóstico prévio de diabetes foram mais propensos a falha no tratamento.	México 2015
Carrión-Torres et al. (10)	– Coorte retrospectiva observacional – Nível IV	– Comparação das características clínicas dos pacientes, tempo de conversão de escarro, presença de cavitação e taxa de cura, duração do tratamento e proporção de alteração do regime de tratamento em 31 pacientes com e 144 sem diabetes.	Existem diferenças nas características demográficas, clínicas e radiológicas em pacientes com tuberculose e diabetes. O tempo de conversão de escarro em negativo é maior em pacientes com diabetes.	Peru 2015
Santana et al. (24)	– Coorte retrospectiva transversal – Nível IV	– 2 189 casos de tuberculose (2004 a 2010) não tratados que, no momento do diagnóstico, tiveram diagnóstico de diabetes autorrelatado ou por critérios diagnósticos.	Os relatórios clínicos de coorte de tuberculose detectaram 135 pacientes diabéticos e 273 não diabéticos. A distribuição de gênero foi semelhante entre os grupos (sexo masculino = 64,4% na tuberculose versus 54,8% daqueles com comorbidade, *P* = 0,11).	Brasil 2016
Junior et al. (23)	– Estudo de coorte prospectivo – Nível IV	– 1 545 pacientes adultos sintomáticos respiratórios examinados de maio de 2010 a setembro de 2011 e selecionados para diabetes.	A prevalência de tuberculose foi de 11,8% (n = 105); 63,1% dos pacientes com sintomas respiratórios apresentaram algum grau de transtorno no metabolismo da glicose.	Brasil 2016
Pereira et al. (17)	– Caso-controle com regressão logística – Nível IV	– 323 casos novos de tuberculose com resultados positivos à baciloscopia e 323 controles.	Média de idade: 38,5 anos (DP = 14,2); em controles, 38,5 (DP = 14,3) anos. 61% eram do sexo masculino. Houve associação entre diabetes e tuberculose (OR = 2,37; IC95%: 1,04 a 5,42).	Brasil 2016
Lacerda et al. (22)	– Epidemiológico, transversal – Nível IV	– 1 984 pacientes com tuberculose de 2001 a 2013 rastreados para diabetes.	A frequência de diabetes em doentes com tuberculose foi de 3,8%, com predomínio de sexo masculino (85%), faixa etária de 40 a 59 anos (75%) e baixa escolaridade (35%).	Brasil 2016
Rocha et al. (16)	– Descritivo, transversal – Nível VI	– 46 pacientes com tuberculose rastreados para diabetes em hospital referência no tratamento da tuberculose.	Prevalência de diabetes: 15%; desses, 80% eram homens e 85% tinham de 20 a 59 anos. 29% dos pacientes desconheciam ter diabetes.	Brasil 2016
Abreu et al. (21)	– Estudo descritivo – Nível VI	– Associação entre 1 090 375 registros de casos de tuberculose e 1 246 137 cadastros de diabetes.	Foram encontrados 24 443 casos de comorbidade, incluindo 3 181 não informados no sistema de registros.	Brasil 2017
Nascimento et al. (26)	– Descritivo, qualitativo – Nível VI	– Análise de 12 entrevistas com gestores e profissionais de saúde para analisar os desafios e potencialidades do manejo integrado de tuberculose e diabetes como política pública.	Os principais desafios detectados foram: cuidado fragmentado; falhas no tratamento diretamente observado; desarticulação entre as esferas de governo e os níveis de atenção; falta de priorização do manejo integrado de tuberculose-diabetes como problemas de saúde pública; tuberculose ainda negligenciada.	Brasil 2017

Outro estudo realizado no Brasil encontrou prevalência de 15% da comorbidade em hospital de referência para tratamento de tuberculose, onde o manejo integrado não era implantado no serviço como protocolo (16). Dados primários também foram utilizados no Chile, encontrando prevalência de comorbidade de 13,6% (17).

### Disparidade de gênero

Os estudos que analisaram a prevalência da comorbidade tuberculose-diabetes concluíram que o sexo masculino é mais afetado (7, 16, 17, 18-21). As maiores prevalências de comorbidade foram relatadas em dois desses estudos, realizados no Brasil, que encontraram 85% e 80%, respectivamente (16, 21).

Dentre os estudos que analisaram a variável gênero, apenas dois (ambos do México) identificaram o sexo feminino como mais afetado (12, 22). Ainda, no México houve similaridade estatística na proporção de homens e mulheres (homens, 64,5% [233/361] versus 70,4% [369/524], *P* = 0,065) (23).

### Profissão/ocupação e local de residência dos pacientes

Alguns estudos apresentaram características sociodemográficas dos pacientes, como idade ou faixa etária (predominando os pacientes mais velhos, acima de 45 anos, 50,7% e 61%) (11, 15); sexo ou gênero (predominando o sexo masculino) (7, 16-21); raça/cor de pele preta ou parda (68,4%) (21) e renda baixa (OR = 1,05 a 2,1) (7, 20). Porém, apenas dois informaram características de local de residência. No primeiro, a probabilidade de morte em indivíduos com tuberculose-diabetes foi maior entre aqueles institucionalizados (OR = 2,69) (14). Contraditoriamente, no segundo estudo os sujeitos com tuberculose-diabetes foram menos propensos a serem institucionalizados (OR = 0,74) (15). Conforme o estudo que trouxe dados referentes a profissão/ocupação, 41% possuíam emprego informal (16).

### Controle glicêmico

Alguns estudos observaram controle glicêmico ineficaz nos pacientes que apresentavam comorbidade (12, 13, 15, 17, 19, 21), porém a maioria utilizou dados secundários para resultados da triagem diagnóstica, como consulta a prontuários. Assim, não houve, nesses locais pesquisados, um rastreamento diagnóstico com dados primários para identificar o controle da glicemia. No México, houve justificativa de recursos limitados para o rastreamento (13). Apenas dois estudos utilizaram o exame de HbA1c para rastreamento do diabetes (22, 23).

Outro estudo relatou que 63,1% dos pacientes com sintomas respiratórios que buscavam cuidados na clínica de referência de tuberculose apresentaram algum grau de transtorno do metabolismo da glicose, definido como HbA1c ≥ 5,7%, indicando um controle glicêmico ruim (22). Pacientes com tuberculose e controle ruim de glicemia tiveram taxas mais altas de falha no tratamento, recaída e morte em relação àqueles com bom controle (24). A rifampicina foi apontada como dificultador do controle glicêmico (12, 22), sugerindo a necessidade de adequação dos fármacos antituberculostáticos em obesos e naqueles com controle glicêmico ruim (25).

Alguns estudos analisaram o conhecimento prévio do diagnóstico de diabetes em pacientes com tuberculose. No Chile, 80% desconheciam ter diabetes (19). No México, o diabetes foi diagnosticado em 19,4% de 70 pacientes, dos quais 16 (22,9%) desconheciam ter a doença (23). No Brasil, um estudo em que a prevalência de diabetes em pacientes com tuberculose foi de 15% relatou que 71% dos pacientes já haviam sido diagnosticados; 29% foram diagnosticados durante a pesquisa (16).

O desconhecimento sobre tuberculose, em pacientes com diabetes, também foi relatado. No México, de 783 pacientes selecionados com diabetes, 38 tinham comorbidade (4,9%) e 11 (28,9%) desconheciam possuir tuberculose (23).

### Registro da comorbidade tuberculose-diabetes

Em relação ao registro de tuberculose e diabetes em prontuários ou fichas eletrônicas de informação, os estudos observaram falhas, ausência de anotações ou subnotificação dos casos registrados (7, 10, 19, 23, 24).

Em relação à tuberculose, um estudo brasileiro identificou que o banco de dados SINAN não incluiu o tipo de diabetes, as características de comorbidades e os resultados de testes de cultura e suscetibilidade a medicamentos (14). No Brasil, o campo “agravo associado à tuberculose” não é de preenchimento obrigatório. Por isso, o registro apresentou-se com grande percentual em branco, o que pode ter prejudicado a análise do estudo. Além disso, o desconhecimento sobre a comorbidade pode ter dificultado a assistência e prejudicado a investigação intradomiciliar de novos casos (20).

No Peru, praticamente nenhum caso de diabetes foi registrado entre os fatores de risco para tuberculose (19). Os casos de comorbidade podem ter sido subestimados por desconhecimento do diabetes (13, 15, 16, 19) e pela negligência observada em relação ao registro da tuberculose (14, 17, 21, 26).

### Custo diagnóstico

O custo financeiro do manejo clínico de indivíduos com tuberculose-diabetes (24, 27) também foi examinado, assim como os recursos limitados na atenção primária (13, 27) para o rastreamento e diagnóstico eficaz do diabetes. O primeiro estudo, do Brasil, relatou que um número maior de encaminhamentos de pacientes para centros de cuidados terciários implica pior prognóstico clínico na associação tuberculose-diabetes, ao mesmo tempo em que a ausência de cuidados especializados para o gerenciamento desses casos no sistema de saúde pode amplificar o custo da atenção à saúde (24).

O segundo estudo, realizado no Peru, demonstrou que 56% da população têm que arcar financeiramente com o custo de serviços de saúde voltados para o tratamento da diabetes e outras doenças crônicas não transmissíveis, ao contrário do programa de tuberculose, que oferece serviços gratuitos, tornando difícil o manejo integrado dessas doenças no contexto do sistema de saúde do país (27).

### Repercussões clínicas no tratamento, cura e mortalidade

Entre os pacientes com diabetes em tratamento para tuberculose, todos tiveram concentrações séricas de pico de isoniazida, rifampicina ou ambas, acima do intervalo esperado. Observou-se também piora na depuração microbiológica, ocasionando atraso na conversão do escarro em negativo (10, 15, 24), com maior probabilidade de falha terapêutica (13, 18).

No Brasil, a proporção de pacientes com esfregaço de escarro inicial positivo foi maior entre o grupo com tuberculose e diabetes do que nos indivíduos sem comorbidade (84,7% vs. 69,1%; *P* < 0,001). Após 30 dias de início do tratamento, os esfregaços positivos foram mais frequentes na presença de tuberculose e diabetes (40,9% vs. 14,7%; *P* = 0,01) (24).

No México, enquanto as taxas de incidência de tuberculose sem diabetes diminuíram, as taxas de comorbidade aumentaram consideravelmente. Os pacientes com tuberculose e diabetes estavam mais propensos a sofrer falência do tratamento, mesmo com (98,39%) tratamento diretamente observado (13).

Um estudo no Peru relatou associação entre diabetes e risco aumentado do desfecho combinado de falha, morte e recaída (27). Ainda no Peru, de 149 pacientes com tuberculose e diabetes e resultados de sensibilidade a drogas, 104 (69,8%) apresentavam tuberculose sensível e 45 (30,2%) tinham tuberculose resistente. Desses, 29 tinham TBMDR (18).

No entanto, as alterações no tratamento dos pacientes com tuberculose e diabetes não refletem, necessariamente, falha no tratamento, pois a falta de um esquema terapêutico para esse tipo de paciente faz que os médicos utilizem diferentes manejos farmacológicos (10, 28). Por outro lado, pacientes sob gestão conjunta, no México, tiveram maior probabilidade de se curar e maior probabilidade de completar o tratamento sem mostrar evidência de falha e sem bacteriologia negativa e padrão em relação aos grupos controles. Pacientes com diabetes em uso de insulina tiveram melhor conversão de escarro (94%) em comparação aos que utilizavam hipoglicemiantes orais (76%). A taxa de recidiva entre os diabéticos foi maior (23,3%) em comparação com não diabéticos (8%) (23).

### Medidas para o manejo integrado

Em relação às evidências científicas acerca do manejo integrado de tuberculose e diabetes e das medidas para sua implementação, observou-se que estão relacionadas à criação de protocolos. Esses protocolos devem enfocar a realização de exames de rotina e triagem bidirecional (16, 23, 28), a utilização da HbA1c como marcador do controle glicêmico (22, 23), o rastreio de tuberculose em serviços de saúde especializados no atendimento à diabetes (12, 13) e o registro de diabetes e tuberculose nos seus respectivos programas (7, 14, 19). Também são importantes as atividades educativas, de prevenção e de acompanhamento dos pacientes, atentando para a especificidade de gênero e idade, juntamente com aspectos sociodemográficos (16, 17, 21).

## DISCUSSÃO

O desenvolvimento de recomendações para o manejo de tuberculose e diabetes, publicadas em 2011, estimulou o aparecimento de projetos-piloto, políticas e discussões sobre a magnitude da comorbidade, desencadeando novas pesquisas sobre mudanças nas diretrizes de cuidados para a prevenção e promoção desses agravos. Evidenciaram-se estratégias para o manejo integrado, aplicado em estudos recentes. Contudo, a aplicabilidade do manejo integrado nos serviços de saúde depende de sua adequação aos modelos de atenção à saúde (protocolos) e às taxas locais de incidência de tuberculose.

Entre os estudos selecionados, 12 (60%) foram classificados como tendo nível de evidência IV, ou seja, apresentam baixa qualidade de evidência científica. Sendo assim, outros estudos precisam ser desenvolvidos na América Latina para que a efetividade do manejo fique evidenciada, permitindo a execução e a discussão de políticas públicas de acordo com a realidade de cada país (9). A proposta para criação e implementação de protocolos de triagem bidirecional tuberculose-diabetes é embasada, dentre outros, na taxa de prevalência e nas repercussões do tratamento.

Em maio de 2014, a Assembleia Mundial da Saúde, da OMS, aprovou a estratégia global para a tuberculose “pós-2015”, que incorpora elementos essenciais para atividades colaborativas para tuberculose-diabetes e destaca a importância de abordar as doenças crônicas como parte da agenda de saúde e desenvolvimento amplo (29). O efeito prejudicial comprovado do diabetes sobre a incidência e os resultados da tuberculose levanta questões importantes sobre a forma como o financiamento e a infraestrutura da tuberculose devem ser usados para diagnóstico e tratamento do diabetes nos países em desenvolvimento, além de revelar inúmeras oportunidades de progresso na assistência e na pesquisa.

O custo da realização de rastreio das doenças em todos os pacientes nos serviços de saúde pode ser um fator dificultador, principalmente nos países em desenvolvimento. Por outro lado, os custos associados ao tratamento da tuberculose na África subsaariana podem ser até 10 vezes a renda anual média para os pacientes na faixa dos 20% mais pobres da população. Para muitas famílias, esses custos são catastróficos (30). Na Tanzânia, o custo mensal com insulina representa 25% do salário mínimo do país. Em Moçambique, o custo anual do tratamento de diabetes com insulina representava 75% da renda *per capita*; em Mali, 61%, incluindo consultas ambulatoriais, internações hospitalares e manejo das complicações da doença (31).

Para aperfeiçoar o diagnóstico de diabetes, a HbA1c deve ser o padrão ouro dos programas, pois avalia os níveis de glicemia ao longo de um período de 2 a 3 meses em vez de apenas em um dia determinado (22). Um estudo encontrou que 84% dos pacientes com tuberculose apresentaram falta do controle glicêmico (32). Outro avaliou que 34% dos pacientes tinham o diabetes controlado adequadamente por meio da HbA1c < 7% (33). A necessidade de desenvolver e avaliar testes diagnóstico e formas mais precisas, rápidas, não invasivas e econômicas de monitoramento – incluindo medidas de glicemia e HbA1c – foi reconhecida em 2011 em reunião de especialistas globais em tuberculose e diabetes (34). Dessa forma, controlar os níveis de glicose reduz o risco de desenvolvimento de tuberculose em pessoas com diabetes (22, 33, 35).

Sobre disparidade entre gêneros, houve maior prevalência de tuberculose-diabetes no sexo masculino (7, 16-21). Diversos fatores contribuem para isso, com destaque para a menor frequência dos homens nos serviços de saúde e os fatores sociais e culturais que influenciam diretamente esse vínculo (16, 17, 21). Assim, esse comportamento representa um desafio para o manejo integrado tuberculose-diabetes.

A profissão/ocupação e local de residência foram abordados por poucos estudos (14-16), embora sejam dados solicitados na ficha do SINAN e relevantes para analisar o perfil dos pacientes com as comorbidades. As divergências entre propensão à morte em indivíduos com a comorbidade nos dois estudos realizados no Brasil (14, 15) podem ser explicadas pela possibilidade de subestimação de casos de diabetes no segundo estudo (15). Dessa forma, é necessário o cuidado em instituições, como abrigos, onde a tuberculose pode não ser tratada adequadamente (14). No caso da tuberculose, reconhecida como “doença da pobreza” (26), a garantia de renda e emprego são possibilidades de modificar esse panorama e estigma.

Outro desafio é a constatação de que a rifampicina dificulta o controle glicêmico dos pacientes por aumentar o metabolismo da maioria dos hipoglicemiantes orais (27, 36). A tuberculose pode provocar uma condição conhecida como “tolerância diminuída à glicose”, que remete a um aumento temporário do nível de açúcar no sangue, gerando um fator de risco para o desenvolvimento de diabetes (22, 35). Pacientes que fazem uso de insulina têm melhor conversão de escarro (94%) em comparação com os que utilizam hipoglicemiantes orais (76%) (35).

No Brasil, em relação ao tratamento farmacológico da tuberculose em pacientes com diabetes, a recomendação é o esquema básico. Porém, naqueles que estão em uso de hipoglicemiante oral, deve-se aumentar a dose e realizar um controle rigoroso da glicemia, que deve ser mantida abaixo de 160 mg/dL. O Ministério da Saúde orienta a troca do hipoglicemiante oral (metformina) por insulina para melhor controle glicêmico devido à interação com a rifampicina. O tempo de tratamento de tuberculose para os pacientes com diabetes em uso de insulina pode ser prolongado (36).

Em outra vertente, alguns estudos (7, 16, 26) consideram que a associação entre tuberculose e diabetes é subvalorizada ou subestimada em função do fato de não ser vista como uma prioridade de saúde pública (28), ou ainda pelo grande número de problemas não esclarecidos relativos à associação (26). Por isso, há necessidade de incentivar pesquisas, com apoio financeiro dos governos locais, especialmente nos países em desenvolvimento.

Ressalta-se que as produções na América Latina e no Brasil precisam ser incrementadas. Por se tratarem de países em desenvolvimento, onde a epidemia do diabetes é crescente e os determinantes sociais (como pobreza, abuso de drogas, baixa escolaridade, segregação e confinamento de indivíduos) têm forte carga, a vulnerabilidade do indivíduo e a permanência da tuberculose representam um desafio ainda maior (10).

Diante do contexto, observa-se um papel fundamental da equipe multidisciplinar no controle desses agravos. Destaca-se a importância das capacitações voltadas a prevenção, promoção e recuperação da saúde dos pacientes, nos três níveis de atenção.

O presente estudo tem limitações, entre elas a escassez de artigos abordando os cuidados específicos aos pacientes com tuberculose e diabetes. Também foi uma limitação a busca limitada a três bases de dados, sem revisão manual de referências ou consulta a documentos oficiais de governos publicados fora da literatura médica e a inclusão apenas de artigos com texto integral de acesso livre.

## Conclusões

O manejo integrado de tuberculose-diabetes desponta como uma ação em saúde capaz de promover benefícios para indivíduos, comunidades e sistemas de saúde, além de potencializar o tratamento e controle dessas condição a um custo financeiramente mais viável do que o tratamento isolado de cada uma delas. São necessárias estratégias para melhorar o acesso aos cuidados em saúde, implementação de prevenção ativa e de esforços coordenados para planejamento e execução de programas, capacitação dos profissionais de saúde e promoção de testes diagnósticos eficazes e acessíveis. Espera-se que o presente estudo possa contribuir para a literatura e estimule as produções científicas latino-americanas, principalmente em áreas que são endêmicas para tuberculose e onde a epidemia de diabetes é iminente.

## Contribuição das autoras.

CVN concebeu o estudo. SMS e CVN coletaram e analisaram os dados e redigiram o manuscrito. Ambas as autoras aprovaram a versão final do manuscrito.

## Agradecimentos.

Agradecemos à Fundação de Amparo à Pesquisa de Minas Gerais (FAPEMIG) pelo apoio financeiro.

## Conflitos de interesse.

Nada declarado pelas autoras.

## Financiamento.

O presente trabalho foi realizado com apoio financeiro da Fundação de Amparo à Pesquisa de Minas Gerais (FAPEMIG) através da concessão da bolsa de pesquisa do Programa de Capacitação em recursos Humanos - PCRH (Processo HBD -00026-18).
